# Editorial: Diabetes and obesity effects on lung function, volume II

**DOI:** 10.3389/fendo.2023.1345489

**Published:** 2023-12-07

**Authors:** Hong Zheng, Weixing Ma, Xiaoqiang Tang, Albert Lecube, Liang-Jun Yan

**Affiliations:** ^1^Department of Basic Theory of Traditional Chinese Medicine, College of Traditional Chinese Medicine, Shandong University of Traditional Chinese Medicine, Jinan, Shandong, China; ^2^Qingdao Municipal Center For Disease Control&Prevention, Qingdao, Shandong, China; ^3^Key Laboratory of Birth Defects and Related Diseases of Women and Children of MOE, State Key Laboratory of Biotherapy, West China Second University Hospital, Sichuan University, Chengdu, China; ^4^Endocrinology and Nutrition Department, University Hospital Arnau de Vilanova de Lleida, Obesity, diabetes and nutrition Research Group (ODIM), Institut de Recerca Biomèdica de Lleida (IRBLleida), Universitat de Lleida, Lleida, Spain; ^5^Department of Pharmaceutical Sciences, UNT System College of Pharmacy, University of North Texas Health Science Center, Fort Worth, TX, United States

**Keywords:** obesity, diabetes, lung function, dyspnea, tuberculosis, lifestyle

Obesity and diabetes have deleterious effects on lung function (1–3). While diabetes and obesity per se can cause pulmonary dysfunction, they can also contribute, as risk factors to the pathogenesis of a variety of pulmonary diseases including tuberculosis (TB) (4), asthma (5), chronic obstructive pulmonary disease (COPD) (6), idiopathic pulmonary fibrosis (IPF) (7), OVID-19 (8), dyspnea (9), and lung cancer (10). In this Research Topic on “*Diabetes and Obesity Effects on Lung Function*” edited by Drs. Tang, Lecube, and Yan, 8 papers were successfully collected that further contribute to our understanding of the mechanisms by which obesity and diabetes impact the above-mentioned pulmonary diseases.

With respect to the effects of type 2 diabetes (T2D) on TB development in diabetic patients, Park et al. evaluated lifestyle changes and risk of TB in T2D patients. The authors found that in diabetic patients, new smokers or consistent smokers exhibit a higher rate of TB development than those who quit or never smoked. Likewise, both consistent heavy alcohol drinkers and heavy drinker quitters exhibit higher TB risk than those who never drank or were new heavy drinkers. Regarding exercise, the authors also found that non-exercisers and exercise-quitters showed a higher TB risk than new exercisers. There is a link between unhealthy lifestyles and increased TB risk in T2D patients, suggesting that changing lifestyles is a good approach for control of TB development in diabetic patients. Additionally, a systemic review and meta-analysis by Rehman et al. have also demonstrated that diabetes is a significant factor for TB treatment failure and the emergence of increased multi-drug resistance caused by diabetic hyperglycemia. However, whether lowering diabetic glucose content can improve TB treatment and retard the development of multi-drug resistance remains elusive. Nonetheless, Meng et al. presented data in this Research Topic that effective glycemic control can mitigate cavity occurrence and increase sputum-negative conversion as well as lesion absorption which are all associated with TB disease, suggesting that good glycemic control in diabetic patients can attenuate TB development and progression.

On T2D effects on dyspnea, Mizab et al. evaluated diabetes effects on dyspnea by conducting surveys using two well-established questionnaires. One was the modified Medical Research Council (mMRC) questionnaire, and the other was the St. George respiratory questionnaire designed for patients who have obstructive airway disease. The authors found that patients with T2D exhibited a higher mMRC score than those who were healthy. Moreover, participants with T2D and mMRC ≥ 2 exhibited a higher HbA1c and also a higher prevalence of kidney disease, demonstrating that diabetes associated with kidney disease contributes to increased sensation of dyspnea.

With respect to T2D as a risk factor for a variety of lung diseases including asthma, chronic obstructive pulmonary disease (COPD), idiopathic pulmonary fibrosis (IPF), and COVID-19, Zhang et al. reviewed the underlying mechanisms of each of these lung disorders and their link to diabetes. The authors presented strong evidence that endothelial microangiopathy is a common pathological mechanism of diabetes and each of the discussed pulmonary disorders. Further understanding of the interactions between T2D and pulmonary disease may provide novel insights into developing new approaches for the management of diabetes and respiratory disorders. Along the line of metabolic issues and lung disease, Malki et al. also performed a systemic review and meta-analysis on a potential link between obesity and lung cancer metastasis. These authors found that obesity is associated with an increase in hospital readmissions of lung cancer patients, demonstrating that obesity is a significant factor that should be considered when it comes to the management of lung cancer and healthcare plans and decision-making. Additionally, with respect to cell death in diabetic pulmonary disorders, Dai et al. reviewed the underlying mechanisms by focusing on non-apoptotic programmed cell death (NAPCD). These NAPCDs include autophagy, necroptosis, ferroptosis, pyroptosis, and copper-induced cell death. The authors pointed out that the formation of advanced glycation end products (AGEs) and their toxicity involving AGE receptors is a common mechanism underlying all these NACPDs that may collectively contribute to diabetic pulmonary dysfunction.

Finally, obesity, in particular, comorbid obesity, is known to contribute to asthma severity (11). Nonetheless, the underlying mechanism remains elusive. Womble et al., using a mouse model of vertical sleeve gastrectomy (VSG) that serves the purpose of bariatric surgery, demonstrated that obesity induced by high-fat diet contributed to the development of glucose intolerance when challenged by intermittent exposures to house dust mites (HDM) that serves as an allergen. This glucose intolerance and HDM-induced asthma were attenuated by VSG. The reason for this improvement is likely due to increased airway hyper-responsiveness induced by SVG in allergic mice. This study therefore shed light on novel approaches for managing airway inflammation and asthma under comorbid obesity conditions.

In summary, obesity and diabetes have a plethora of detrimental effects on many lung diseases including TB, asthma, IPF, COVID-19, COPD, dyspnea, and lung cancer. Non-programmed cell death and endothelial microangiopathy may be therapeutic targets as they seem to be common mechanisms underlying the deleterious effects of obesity and diabetes on these lung diseases. Proactive lifestyle changes or bariatric surgery toward an effective control of glycemia may mitigate the deleterious effects of obesity and diabetes on some, if not all, of the lung disorders discussed in this Research Topic.

## Author contributions

HZ: Writing – original draft, Writing – review & editing. WM: Writing – original draft, Writing – review & editing. XT: Writing – review & editing. AL: Writing – review & editing. L-JY: Writing – original draft, Writing – review & editing.
